# Emerging strategies in *BRCA*-positive pancreatic cancer

**DOI:** 10.1007/s00432-018-2666-9

**Published:** 2018-05-18

**Authors:** Adam Kowalewski, Łukasz Szylberg, Michał Saganek, Wojciech Napiontek, Paulina Antosik, Dariusz Grzanka

**Affiliations:** 1 Department of Clinical Pathology, Collegium Medicum in Bydgoszcz, Nicolaus Copernicus University in Torun, Sklodowskiej-Curie Str. 9, 85-094 Bydgoszcz, Poland; 2 Department of Pathology, Military Clinical Hospital, Powstańców Warszawy Str. 5, 85-681 Bydgoszcz, Poland

**Keywords:** Pancreatic cancer, BRCA, Mutation, Platinum, PARP inhibitor, Targeted therapy

## Abstract

**Purpose:**

We propose a treatment algorithm for PDAC with particular emphasis on *BRCA1* or *2* mutation-positive patients. Pancreatic ductal adenocarcinoma (PDAC) is one of the deadliest diseases in the United States and Europe. *BRCA1* and *BRCA2* are among the most common of the known genetic mutations involved in familial PDAC. The optimal chemotherapy regimen to use for *BRCA1* or *2* mutation carriers with PDAC is not yet established. As new treatment options emerge, algorithms must balance the need to give the best drugs first with ensuring that there are still beneficial options available for later.

**Methods:**

We conducted a review of the literature for data on possible therapies in *BRCA-*positive and *BRCA*-negative pancreatic cancer.

**Results:**

There is accumulating evidence of increased sensitivity to platinum-based therapy and poly-ADP-ribose polymerase inhibitors (PARPi) in *BRCA*-associated PDAC. There are no studies relating to borderline *BRCA*-associated PDAC and, therefore, same treatment as for sporadic PDAC seems appropriate. Treatment of unresectable PDAC varies depending on stage of the disease. Patients with *BRCA*-associated locally advanced PDAC can benefit from targeted therapy with PARPi (olaparib) as a second-line therapy after antimetabolite treatment failure. Patients with unresectable metastatic *BRCA*-positive PDAC may benefit from platinum-based therapy.

**Conclusion:**

Targeted therapies are shifting the treatment paradigms and increasing survival for patients with PDAC, a group that used to have a grim prognosis.

## Introduction

Pancreatic cancer is one of the deadliest diseases in the United States and Europe, and the fourth leading cause of cancer-related death (Von Hoff et al. 2013). Of all pancreatic cancers, 95% are adenocarcinomas of the ductal epithelium. The 5-year survival rate for patients with stage IA exocrine pancreatic cancer is about 14%, while in IV stage it does not exceed 1% (Surgery for Pancreatic Cancer 2016). The identification of molecular mechanism associated with pancreatic carcinogenesis is of utmost importance for understanding the nature of pancreatic cancer (Beger et al. 2004). A family history of pancreatic cancer is found in 5–10% of pancreatic cancer patients (Klein 2012). Pancreatic ductal adenocarcinoma (PDAC) occurs especially in families with ovarian or breast cancers (Golan et al. 2014).

*BRCA1* and *BRCA2* are the most common of the known genetic mutations involved in familial pancreatic cancer (Leung and Saif 2013). Family studies have demonstrated that both *BRCA1* and *BRCA2* mutation carriers have an increased risk of developing pancreatic cancer (Beger et al. 2004). In case of *BRCA1* mutation carriers the relative risk for pancreatic cancer is 3.1 and 6.6 in relatives of *BRCA2* mutations carriers (Iqbal et al. 2012). Somatic genomic analysis has identified four specific subtypes of pancreatic adenocarcinoma stable, locally rearranged, scattered and unstable (Biankin and Maitra 2015).

*BRCA1* and *BRCA2* mutations are related to the unstable subtype exhibiting a unique mutational signature reflecting DNA damage repair deficiency (Golan et al. 2014). The emergence of molecular tests allows us to tailor treatment strategies based on the presence of driver mutations.

Patients with breast or ovarian *BRCA*-related cancer now benefit from targeted therapies in the first line and beyond. However, the optimal chemotherapy regimen to use for *BRCA1* or *2* mutation carriers with PDAC is not yet established. As new treatment options emerge, algorithms must balance the need to give the best drugs first with ensuring that there are still beneficial options available for later. This paper discusses treatment approaches for patients with unresectable PDAC, especially in patients with *BRCA*-related PDAC.

### *BRCA* mutation

Both *BRCA1* and *BRCA2* are tumor suppressor genes (Leung and Saif 2013).

Mutations of these genes may play a pivotal role in tumor genesis and cancer progression (Zhu et al. 2017).

Prevalence of *BRCA1* and *BRCA2* mutations fluctuates between 1 in 300 and 1 in 800 and depends on the population. There are more than 2000 known mutations in *BRCA1 or 2* genes. In some populations founder mutations are the most frequent. For example, about 2.5% of the general Ashkenazi Jewish population will harbor mutation of *BRCA1*. The founder mutations also occur in Northern, Western and Eastern Europe. For that time penetration is variable and not expressly understood (Paluch-Shimon et al. 2016).

Cells with reduced activity of BRCA1 or 2 proteins accumulate double-strand breaks that cause genomic instability and consequently increased predisposition to malignant transformation and progression. Somatic, biallelic inactivation of the *BRCA1 or 2* genes confers sensitivity to inhibition of poly(ADP-ribose)-polymerase (PARP), an enzyme involved in base excision repair (Beger et al. 2004).

Cell with dysfunction in *BRCA1 or 2* genes use less accurate mechanism to repair double-strand breaks which increase the probability of cell death (Solomon and Everett 1990).

## Discussion

The optimal chemotherapy regimen to use for *BRCA1 or 2* mutation carriers with pancreatic adenocarcinoma is not established. Ryan et al. suggest using the same chemotherapy regimens in the adjuvant setting as are used for non-mutation carriers (Ryan et al. 2018). However, there is accumulating evidence of increased sensitivity to platinum-based therapy and poly-ADP-ribose polymerase inhibitors (PARPi) in *BRCA*-associated PDAC (Ryan et al. 2018).

Among tested agents these two represent a promising alternative for BRCA-associated PDAC. Seven studies (Golan et al. 2014; Oettle et al. 2014; Kaufman et al. 2015; Wu et al. 2015; Hurt et al. 2017; Kobayashi et al. 2017; Xu et al. 2017) that investigated a predictive role of *BRCA1 or 2* mutation among patients with pancreatic cancer are summarized in Table 1. Most of them showed significantly enhanced response to DNA damage-related agents.

**Table 1:** Studies investigating a predictive role of BRCA1 or 2 mutation among patients with pancreatic ductal adenocarcinoma (PDAC)

	Characteristic of patients	No. of patients	Type of therapy	Results	References
Locally advanced PC	Patients with advanced PC	36	Capecitabine + RT	OS = 17.6 months PFS = 12 months	Hurt et al. (2017)
		38	Gemcitabine + RT	OS = 14.6 months PFS = 10.4 months	
	Patients with *BRCA1 or 2* mutation and advanced PDAC, after gemcitabine treatment failure	23	PARPi (olaparib)	OS = 9.8 months PFS = 4.6 months	Kaufman et al. (2015)
	Patients with advanced PC after gemcitabine treatment failure	76	Oxaliplatin + FA + FU	OS = 5.9 months PFS = 2.9 months	Oettle et al. (2014)
		84	FA + FU	OS = 3.3 months PFS = 2.0 months	
Metastatic PC	Patients with *BRCA1 or 2* mutation and stage III/IV PDAC	22	Platinum based adjuvant therapy	OS = 22 months	Golan et al. (2014)
		21	Non-platinum therapy	OS = 9 months	
	Patients with metastatic PC	83	Gemcitabine + paclitaxel	OS = 9.2 months PFS = 5.5 months	Xu et al. (2017)
	Patients with metastatic PC after gemcitabine treatment failure	18	FOLFIRINOX	OS = 9.8 months PFS = 2.8 months	Kobayashi et al. (2017)
	Patients with metastatic PC after gemcitabine treatment failure	17	Lapatinib + capecitabine	OS = 5.2 months PFS = 2.6 months	Wu et al. (2015)

*PC* pancreatic cancer, *PDAC* pancreatic ductal adenocarcinoma, *RT* radiotherapy, *PARPi* poly(ADP-ribose)-polymerase inhibitor, *FA* folinic acid, *FU* fluorouracil, *OS* overall survival, *PFS* progression-free survival

## Platinum agents

In three studies (Oettle et al. 2014; Kaufman et al. 2015; Hurt et al. 2017), there was reported significantly improved OS and response to platinum-based treatment in *BRCA-*positive PDAC. Platinum-based anticancer drugs bind directly to DNA, causing DNA double-strand breaks. Therefore, cells that lack BRCA1 or BRCA2 have a deficiency in the repair of DNA double-strand breaks (Lohse et al. 2016).

## PARP inhibitors

The PARP enzymes play critical roles in DNA damage detection and repair (de Bono et al. 2017).

PARP1 is a protein that is important for repairing single-strand breaks. If the breaks persist unrepaired until DNA is replicated, then the replication itself can cause double-strand breaks to form. PARP1 inhibitors cause multiple double-strand breaks to form in this way, and in tumors with *BRCA1 or 2* mutations, these double-strand breaks cannot be efficiently repaired, leading to death of the cells (Vinayak and Ford 2010).

## Therapy

### Resection

In the early stage of PDAC, surgery offers the only realistic chance for recovery (Surgery for Pancreatic Cancer 2016). Regardless of the use of platinum compounds or PARPi, the prognosis of surgically resectable *BRCA*-associated PDAC is no different than that of sporadic PDAC (Golan et al. 2017). There are no studies relating to borderline *BRCA*-associated PDAC and, therefore, same treatment as for sporadic PDAC seems appropriate (Lopez et al. 2014). Treatment of unresectable PDAC varies depending on stage of the disease. In the following, we separate locally advanced from metastatic tumors.

## Unresectable locally advanced *BRCA*-positive PDAC

### First-line therapy

There are no studies confirming the greater efficiency of any alternative therapy in patients with unresectable locally advanced *BRCA*-related PDAC as a first-line therapy. Antimetabolites appear to be the best option as a first-line therapy. Hurt et al. tested the activity and safety of gemcitabine-based and capecitabine-based chemoradiation for locally advanced pancreatic cancer. mOS was 17.6 months among patients treated with capecitabine-based chemoradiation vs 14.6 months among patients with gemcitabine-based chemoradiation. Also mPFS was higher in group treated with capecitabine-based chemoradiaton (12.0 vs 10.4 months in group treated with gemcitabine-based chemoradiation) (Hurt et al. 2017).

### Second-line therapy

Patients with *BRCA*-associated locally advanced pancreatic cancer can benefit from targeted therapy with PARP inhibitor (olaparib) as a second-line therapy after antimetabolite treatment failure. Leung and Saif described an example of two patients with *BRCA2*-associated pancreatic cancer treated with PARP inhibitors. Patients achieved partial or complete response with non-significant side effects (Leung and Saif 2013). The distinct sensitivity of cancerous cells to PARPi (olaparib) was also observed in study of Lowery et al. (2011). Among 16 cases, 11 had *BRCA2*-associated PDAC. Three out of four patients receiving PARPi and five out of six patients receiving platinum-based chemotherapy demonstrated an initial radiographic partial response. Unfortunately, each patient treated with a PARP inhibitor experienced progression of disease after several months of therapy. It was probably caused by acquired resistance to PARP inhibition.

Similar study showed that tumor response rate for patients treated with PARPi was 21.7%. OS in this group was 9.8 months and PFS was 4.6 months. Severe side effects (grade 3 or 4) were observed in 30.4% of patients. There was no significant difference in response rate for those treated previously with platinum or between *BRCA1* and *BRCA2* mutation. Type of mutation appeared to be not as important as previously suspected. Moreover, prior platinum treatment does not improve patients’ outcomes (Kaufman et al. 2015). Oettle et al. used oxaliplatin, folinic acid (FA) and fluorouracil (FU) also after gemcitabine therapy failure. The data regarding *BRCA* mutation are not applicable. OS in this group was 5.9 months and PFS was 2.9 months. Severe side effects (grade 3 or 4) were observed in 43% of patients (Oettle et al. 2014).

### Third-line therapy

There is no established third-line therapy for patients with *BRCA*-positive PDAC. We suggest using the standard second-line treatment. Treatment with oxiplatine together with FA and FU as a second line, results in OS of 5.9 months (Oettle et al. 2014).

## Unresectable metastatic *BRCA*-positive PDAC

### First-line therapy

Patients with unresectable metastatic *BRCA*-positive PDAC may benefit from platinum-based therapy. Golan et al. compared platinum-based therapy vs non-platinum chemotherapies in patients with *BRCA*-positive pancreatic cancer in stage III/IV. Median overall survival (mOS) in patients treated with platinum agents was higher comparing to those treated with non-platinum chemotherapies (22 vs 9 months, *P* = 0.039). This study also showed that probability of OS in patients treated with platinum is 0.7 in 12 months and 0.16 in 36 months. Probability of OS in patients treated with non-platinum chemotherapy is 0.26 in 12 months and 0.07 in 36 months (Golan et al. 2014).

### Second-line therapy

There is no established second-line therapy for patients with *BRCA*-positive PDAC after platinum-based therapy. Therefore, treatment with gemcitabine with paclitaxel or erlotynib or capecitabine is a reasonable option. Xu et al. conducted a study using gemcitabine with paclitaxel in patients with metastatic pancreatic cancer. In this study, mOS was 9.2 months and median progression-free survival (PFS) was 5.5 months. The most common grade ≥ 3 adverse events were leukopenia (35%), neutropenia (34%), anemia (15%), thrombocytopenia (10%), and fatigue (13%) (Xu et al. 2017).

### Third-line therapy

There is no established third-line therapy for patients with *BRCA*-positive PDAC. Selection of the therapy after gemcitabine treatment failure depends on patient’s condition and life expectancy. Kobayashi et al. studied the effectiveness of FOLFIRINOX in patients with metastatic pancreatic cancer after gemcitabine treatment failure. Among 18 patients receiving FOLFIRINOX, mOS was 9.8 months and PFS was 2.8 months (Kobayashi et al. 2017). Because of high rate of serious side effects (78%), such treatment is recommended especially in patients in good health. Wu et al. studied the effectiveness of lapatinib and capecitabine in patients with metastatic pancreatic cancer after gemcitabine treatment failure. mOS and PFS was lower than after FOLFIRINOX (5.2 vs 9.8 months and 2.6 vs 2.8 months, respectively). However, serious side effects were observed only in 18% of patients (Wu et al. 2015)⁠. This is why lapatinib and capecitabine seem the most accurate in patients with serious side effects after gemcitabine treatment.

## Conclusions

**Fig. 1:**
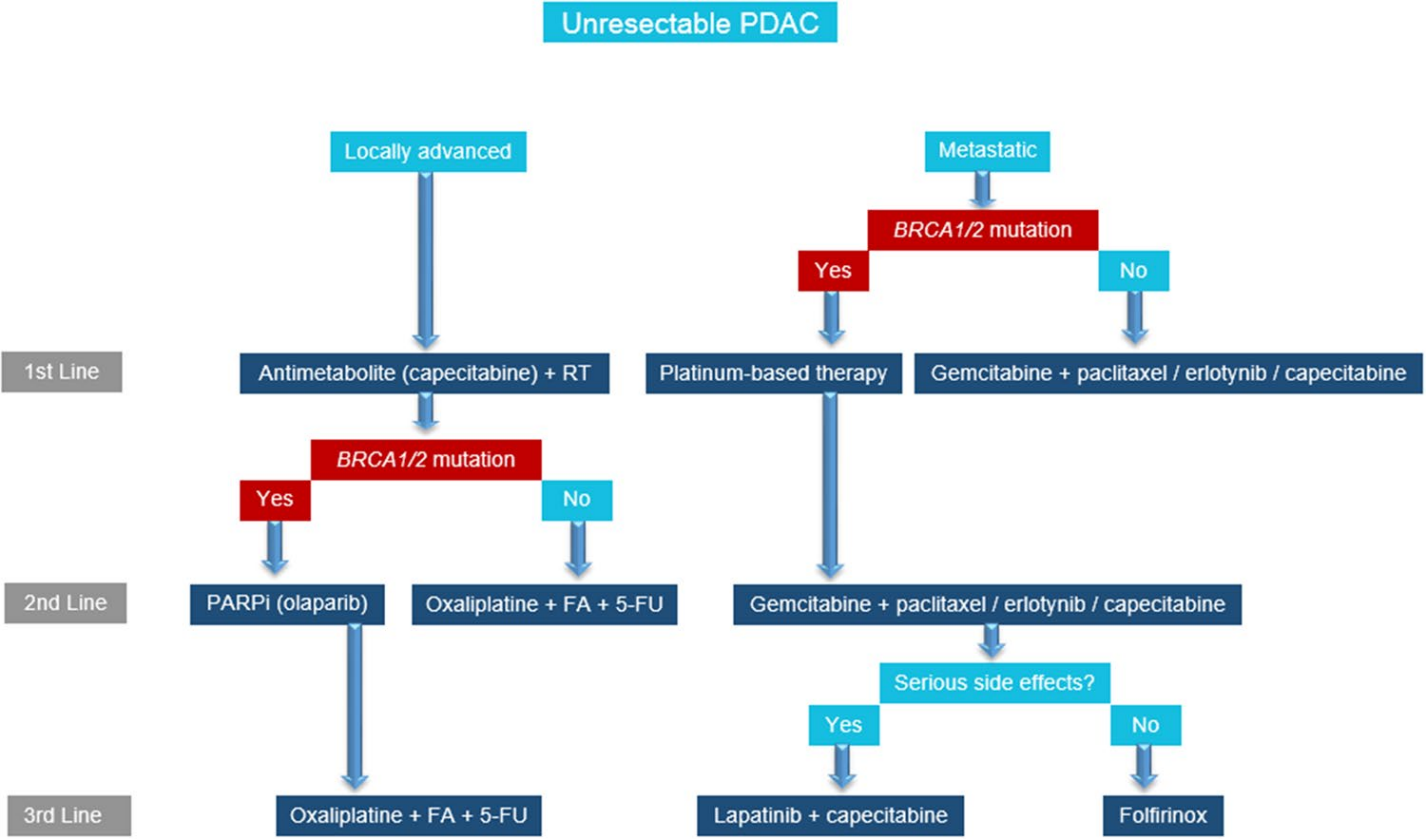
Treatment algorithm for pancreatic ductal adenocarcinoma (PDAC) patients. BRCA1 or 2 mutation determines the optimal therapy

Our understanding of PDAC mutations and their contribution to therapeutic efficacy is expanding. The treatment selection is complex, with new target therapies being developed. Because *BRCA1* and *BRCA2* mutations are relatively rare in the general population, testing should be performed especially when the person’s individual or family history suggests the possible presence of a harmful mutation in *BRCA1* or *BRCA2*. For patients with locally advanced PDAC with *BRCA1* or *2* mutation who have progressed on capecitabine, new treatment options to improve survival include PARP inhibitors such as olaparib. For patients with metastatic PDAC with *BRCA1* or *2* mutation, platinum-based therapy can lead to significant improvements in survival. Targeted therapies are shifting the treatment paradigms and increasing survival for patients with PDAC, a group that used to have a grim prognosis. We propose a treatment algorithm for PDAC with particular emphasis on *BRCA1* or *2* mutation-positive patients (Fig. 1).
